# High throughput sequencing technology facility research of genomic modification crop cultivation influencing soil microbe

**DOI:** 10.3389/fpls.2023.1208111

**Published:** 2023-05-31

**Authors:** Jinyan Jiang, Xin Hu, Xincheng Ji, Haoming Chen

**Affiliations:** School of Environmental and Biological Engineering, Nanjing University of Science and Technology, Nanjing, China

**Keywords:** genetic modified crop, soil microbe, high throughput sequencing technology, microorganisms, crop

## Introduction: the risk in the GM crop cultivation influencing soil microbe and new generation sequencing technologies

The safety of transgenic crops is a hot spot concerned deeply worldwide. Although transgenic crops have great advantages improving agricultural production, their potential impacts on soil safety and soil microorganisms need to be further studied. As an important place for material circulation and energy transformation in the ecosystem, soil is sensitive to changes in the external environment, and soil microbes has been recognized as one of the early warning indicators of ecosystem changes. Soil microorganisms are mainly composed of bacteria, fungi and actinomycetes. Last decades, with the development of modern biotechnology, the main research methods of soil microbial community include microbial plate culture method, Biolog microplate method, biomarker method and the emerging new technologies of high-throughput sequencing.

## Technologies monitoring soil microbe and its transmission: from the classic tech to the NGS

Molecular detection technology is highly sensitive to changes in microbial community structure and can detect even small differences in bacterial composition. It has played a certain advantage in exploring microbial community diversity, particularly when identifying new microbial species. Classical methods include fluorescence *in situ* hybridization (FISH), DNA microarray, single strand conformation polymorphism (SSCP), restriction fragment length polymorphism (RFLP), amplified ribosomal DNA restriction analysis (ARDRA), amplified fragment length polymorphism (AFLP), denaturation/temperature gradient gel electrophoresis (DGGE/TGGE), etc. Among them, DGGE technology is the most widely used method in the study of soil microbial community. These molecular biological methods have the advantages of high sensitivity and no need to cultivate microorganisms, which can better reflect the real situation of soil microorganisms. However, the disadvantages are also obvious. For example, the microbial communities that can be detected in DGGE are usually colonies with significant dominance, and the same band may be shared by multiple microbial species (Ansorge, 2009).

With the rapid development of molecular biology research, especially the wide application of next-generation sequencing (NGS) technology, the research scope and depth of soil microbiology have greatly improved (Nielsen and Wall, 2013; Ranjard et al., 2013). High throughput sequencing technologies mainly include 454 pyrophosphate, ABI SOLiD, Illumina Hiseq2000 and Illumina Miseq, Hecos Heliscope and PacBioSequel sequencing platforms. Up to date, these high-throughput sequencing technologies have gradually replaced DGGE technology, especially the third-generation sequencing technology characterized by single molecule and long reading length, and gradually become the main analytical methods of microbial community research (Fahner et al., 2016). For example, PacBio 16S amplification sequencing technology firstly corrects, filters and removes the chimeric sequences from the amplification and sequencing results (raw reads) of bacterial 16S rRNA genes to obtain valid data (clean reads), then bioinformatic analysis clusters the clean reads into OTUs, and annotates them with species to obtain taxonomic information. Next, Qiime and other software were used to calculate the observed OTUs, Chao1, Shannon, Simpson and other alpha diversity indexes, calculate the Unifrac distance, construct PCoA and UPGMA clustering trees, and analyze the differences between groups. Finally, Anosim, MRPP, ADONIS, t-test and other statistical analysis methods were used to test the difference significance of species composition and community structure among groups.

Macro genome sequencing is new research strategy based on NGS. It takes the entire microbial community in a specific habitat as the research object, and directly extract environmental sample DNA, and obtain the total amount of environmental microbial gene information via high throughput sequencing technology (Tringe et al., 2005). Unlike other high-throughput-sequencing strategy, clean reads need to be assembled by Metagenome to annotate common functional databases, resistance gene databases, and a series of advanced information analysis (such as CCA/RDA analysis, copy number variation analysis, etc.). At the same time, it can also be combined with environmental factors, pathological indicators or special phenotypes for in-depth correlation analysis. Macro genome sequencing technology can help avoid experimental errors due to microbial differences caused by the environment. This technology provides a comprehensive microbial profile in the samples, including their composition and interaction. The resulting data can be used for more in-depth research, such as analyzing metabolic pathways and gene function at the molecular level (Mende et al., 2012). Macro transcriptome sequencing refers to the study of the transcription and regulation of all genomes in a specific environment and period of life groups at all levels. Compared with macro genome sequencing, it takes all RNA in the ecological environment as the research object to study the changes of complex microbial communities at the transcription level, which is another strategy to tap potential new genes (Vieites et al., 2009). All of the cases mentioned above, and the others were listed in **Table 1**.

**Table 1:** Apply high throughput sequencing technologies to analyze the microbe in GM crop cultivation field.

Crop	Exogenous gene	References
Maize	*Bt*	(Zeng et al., 2014)
Maize	*CrylAb*	(Ondreičková et al., 2014)
Maize	*Bt*	(Cheeke et al., 2015)
Cotton	*CrylAb/lAc*	(Xie et al., 2017)
Maize	*Bt*	(Zeng et al., 2019)
Maize	*Bt*	(van Wyk et al., 2017)
Rapeseed	*Glufosinate tolerant*	(Tang et al., 2019)
Soybean	*Glyphosate resistant*	(Girgan et al., 2020)
Soybean	*EPSPS*	(Lu et al., 2018)
Rice	*Bar*	(He et al., 2019)
Maize	*EPSPS*	(Wen et al., 2019)

**Figure 1:**
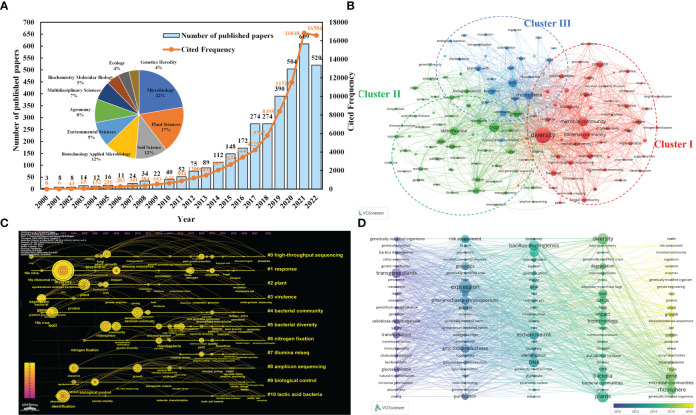
**(A)** Publications, citations performance and the top ten research fields in terms of number of documents. **(B)** Keywords co-occurring network. The main keywords of the three clusters can be divided into the research between soil ecological environment and microbial community/diversity (Cluster I red), the research between microorganisms and crop disease control based on gene level (Cluster II green), and the research between plant root soil microbial characteristics and plant growth (Cluster III blue). The key words “microbial communities” (425), “crop crop” (450) and “high-throughput sequencing” (151) are highly correlated. It can be seen that high-throughput sequencing technology has occupied a place in the study of the interaction mechanism between microorganisms and crops. **(C)** keyword clustering atlas of “High throughput sequencing technology”, “microorganisms” and “crops”. #0 (high-throughput sequencing) is closely connected with # 1 (response), # 2 (plant), # 3 (environment), and # 4 (bacteria community) respectively. Illumina MiSeq (# 7) and cluster # 8 (amplicon sequencing) are commonly used in high throughput technology. **(D)** keywords timeline visualization of “Microorganisms” and “Genetically Modified Crops” from WOS during 2000.01–2022.11. From 2014 to 2018, “GMOs”, “expression”, “plants”, “diversity”, “bacteria”, “gene”, “transgenic plants”, “rhizosphere”, “DNA” and “resistance” appeared frequently.

The bibliometric studies with the keywords "high-throughput sequencing technology", "microorganisms" and "crops" provide further evidence of the trend towards high-throughput sequencing technology, and the retrieval formula was TS= (“microbial” OR “microorganism*” OR “microbe*” OR “microbial community” OR “bacteria” OR “bacterial” OR “fungus” OR “fungi”) AND TS= (“NGS” OR “next generation sequencing” OR “high throughput sequencing technologies” OR “high throughput” OR “sequencing” OR “454 pyrophosphate” OR “ABI SOLiD” OR “Illumina Hiseq2000” OR “Illumina Miseq” OR “Hecos Heliscope” OR “PacBioSequel”) AND TS= (“crop*”). Research exploring the mechanisms of crop-microbe interactions using high-throughput sequencing technologies has generally shown a steady growth trend over the past 22 years (2000.1.1-2022.11.30, 3411 research papers, reviews, conference papers, etc. from Web of Science). The total number of citations up to 77776 times also shows that this research direction has received high attention (**Figure 1A**). Meanwhile, this area of research has piqued the interest of scholars across a range of fields, with the highest interest in microbiology (22%), plant science (17%), soil science (12%) and biotechnology and applied microbiology (12%). The Vosviewer software analysis yielded a co-occurrence network and clustering distribution of the most popular keywords in the field (40 occurrences, 136 nodes) (**Figure 1B**).

The keyword (K) clustering atlas (LLR algorithm: log likelihood ratio, purple and yellow representing the first and latest time respectively, g-index K =15) found that High-throughput technology has gradually become the main means of studying soil microorganisms and plants, and has been widely used in crop variety improvement, plant stress resistance, disease and pest resistance, toxicity resistance, biological gene transformation and other aspects (**Figure 1C**). Moreover, the high-rate appearance of keywords in recent years indicated that transgenic crops affecting soil enzyme activity (e.g., cellobiose dehydrogenase, glucose-hydrogenase, cellobiose dehydrogenase, etc.), risk assessment and the study of soil microbial community structure and diversity have become hot spots between GM crops and microorganisms (**Figure 1D**).

## High throughput sequencing technologies facilitating soil microbe identification in the land of GM crop cultivation

### Insect resistant transgenic crops

Insect resistant genes that have been transferred into crops including: Bacillus thuringiensis toxin protein gene, protease inhibitor gene, amylase inhibitor gene, foreign lectin gene, ribosome inactivating protein gene and pea lipoxygenase gene. At present, the *Bt* and *CpT1* gene are the most popular in the research of evaluating the impact of insect resistant transgenic crops on non-target soil microorganisms (YJ et al., 2014). The high-throughput sequencing results of Liang et al. showed that there were no significant differences in rhizosphere soil bacterial richness and diversity index and community structure between *Cry1Ie* transgenic maize and non-transgenic maize, while principal component analysis (PCA) showed that there were significant differences between seedling, flowering and maturity stages or between different test years (Jingang et al., 2018). The bacterial species diversity and community structure in rhizosphere soil of Bt maize and non-Bt maize were studied by Illumina MiSeq sequencing technology (van Wyk et al., 2017). The results showed that there were significant differences in bacterial alpha diversity and community composition between the two groups. Proteobacteria and Acidobacteria were the dominant bacteria in Bt maize rhizosphere soil, while actinobacteria were the dominant bacteria in non-Bt maize rhizosphere soil. Further study showed that there were significant differences in the contents of organic carbon and nitrate and the activities of acid phosphatase and β-glucosidase in rhizosphere soil between Bt maize and control. These differences in soil physical and chemical properties and related enzyme activities may be the main reasons for the differences in bacterial community structure.

### Herbicide-resistant genetically modified crops

Herbicide-resistant GM crops are the most widely grown crops in the world, even more than insect-resistant GM crops containing Bt genes (Kwon and Kim, 2001). Glyphosate and glyphosate resistance genes are the most commonly used genes in transgenic crops with herbicide resistance. Among them, there are 7 anti-glyphosate genes, which are *cp4 epsps*, *gat4601*, *goxv247*, *epsps*, *epsps grg23ace5*, *mepsps*, *2mepsps*, and anti-glyphosate genes *bar* and *pat*. Similar to the results of studies on insect-resistant GM crops, there are also two kinds of results in studies evaluating the effects of herbicide-resistant GM crops on soil microbial ecosystems. Most of the results showed that the introduction of herbicide resistance genes into crops had no specific effects on the soil microbial community, but was mainly influenced by the seasons and plant development. Tang et al. adopted 16S rRNA gene high-throughput sequencing technology and found that phosphonate resistant rapeseed had no significant effect on the alpha diversity of the rhizosphere bacterial community, and the dominant bacteria with high relative abundance were *Proteobacteria*, *Bacteroides*, *Acidobacteria*, *Blastomonas* and *Actinomycetes* (Tang et al., 2019). These dominant microbes were highly present in transgenic and non-transgenic recipient rapeseed treated with phosphate-oxyphosate, respectively, but there were significant differences in different growth stages (seedling, bolting, flowering and maturity). Moreover, some studies have shown that over a long period of time, some herbicide-resistant transgenic crops can affect the relative abundance of some microorganisms in soil, thus changing the composition of microbial community. Babujia et al. conducted 10 years of field monitoring on glyphosate-resistant transgenic soybeans (Babujia et al., 2016). Although the introduction of glyphosate-resistant genes did not affect the yield of soybeans, metagenomic sequencing analysis found that actinomycta and acidobacteria were abundant in the bacterial community of conventional soybean rhizosphere soil. The relative abundance of Proteus, Firmicutes and green algae was higher in transgenic soybean, and the abundance of sequences related to protein metabolism and cell division cycle were also higher. He et al. conducted Illumina MiSeq sequencing on the 16S rRNA gene of bacteria at maturity stage and found that there was no significant difference in soil bacterial diversity index between BAR-gene transgenic rice and conventional rice; but there were significant differences in the abundance of individual genera (such as *Cerococcus*) between transgenic japonica rice and conventional rice (He et al., 2019). The content of anaerobic bacteria in soil of transgenic japonica rice was significantly higher than that of conventional rice.

## Prospect

Soil microorganisms are crucial to the soil ecosystem and agricultural production. The introduction of foreign genes may affect plant metabolism and root secretion production, thus changing soil microecological environment. Therefore, we need to carry out in-depth risk assessment of GM crops on soil ecosystems. The core of soil ecosystem security risks of GM crops is to assess whether the cultivation of GM crops will change the function of soil ecosystems. So far, a large number of studies have evaluated the potential impact of transgenic crops on the presence of soil microorganisms. However, due to the different objects (including exogenous genes and plant species), environment and experimental methods in these studies, the obtained data are analyzed in different ways. In addition, most of the research results are based on the cultivation period of 1-2 years, so the results are bound to show some differences. With the development of molecular breeding, the development of transgenic crops with multiple trait overlays has not only made research more complex, but also posed new challenges for environmental safety assessment and research. Due to the above limitations of the current research, we should pay more attention to the correlation between the composition and function of soil microbial community structure and its response to the natural changes of soil system (such as season, climate, crop rotation, heavy metal pollution and pesticide use) in the future, so as to assess the impact of GM crops on soil microorganisms more comprehensively and accurately.

## Author contributions

JJ, HC, XH, and XJ conceived and wrote the paper. All authors contributed to the article and approved the submitted version.
